# Light-driven biosensor for the rapid and selective detection of hypoxia-inducible factor-prolyl hydroxylase domain inhibitors in aqueous media and saliva

**DOI:** 10.1007/s00604-025-07579-y

**Published:** 2025-10-02

**Authors:** Rebecca L. Houston, Godwin Ayoko, Emad L. Izake

**Affiliations:** School of Chemistry and Physics, Faculty of Science, Queensland University of Technology (QUT), 2 George Street, Brisbane, QLD 4000 Australia

**Keywords:** PHD2 biosensor, SERS, Light-driven functionalisation, Plasmonic nickel foam, Cauliflower-shaped gold nanostructures, HIF inhibitors, Doping control

## Abstract

Hypoxia-inducible factor-prolyl hydroxylase domain inhibitors (HIF-PHI) are to increase the production of EPO and haemoglobin in patients with chronic kidney disease. HIF inhibitors such as Molidustat and Vadadustat are used as doping agents in racing sports. We present new sensitive and selective materials for the biosensing of Molidustat and Vadadustat in aqueous solution and saliva. Plasmonic nickel foam was synthesised using a reproducible chemical method that deposits forests of closely packed cauliflower-shaped gold nanostructures on the nickel surface. The plasmonic material showed a high enhancement factor of Raman signals (1.09 × 10^7^). Therefore, it was used as a sensor for the detection of HIF inhibitors by surface-enhanced Raman spectroscopy (SERS). A target-specific extractor chip for HIF-PHI was fabricated by functionalising the plasmonic nickel foam with prolyl hydroxylase domain-containing protein 2 (PHD2) molecules. The surface functionalisation was carried out by a green synthesis process that utilises deep UV LED light to convert the PHD2 molecule into a bio-thiol that attaches to the plasmonic nickel foam via Au–S bonds. Using the new SERS sensor and extractor chip, Molidustat and Vadadustat were quantified in aqueous solution down to 0.01 µg/L. The new materials were also utilised for the rapid screening of HIF-PHI in spiked saliva samples. The new materials and light-driven surface functionalisation process can be easily adopted to develop new screening methods for multiple biomolecules (e.g. drugs, proteins, disease biomarkers, environmental toxins) for many applications (e.g. therapeutic drug monitoring, doping control, clinical diagnostics environmental monitoring).

**Graphical Abstract:**

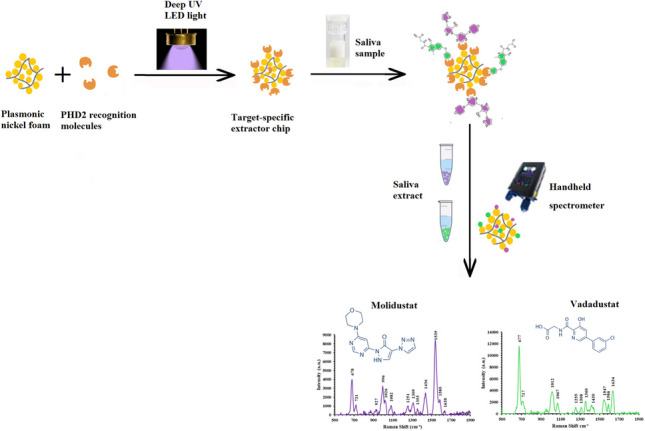

**Supplementary Information:**

The online version contains supplementary material available at 10.1007/s00604-025-07579-y.

## Introduction

Prolyl hydroxylase domain 2 (PHD2) inhibitors (also known as dustats) are a class of pharmaceutical compounds that induce the endogenous erythropoietic system to increase erythropoiesis under normoxic conditions [1]. Therefore, the use of these compounds increases the production of red blood cells and the oxygen supply in patients particularly those with anaemia related to chronic kidney disease (CKD) [1]. Due to this property, athletes are attracted to use dustats such as Molidustat and Vadadustat to increase aerobic exercise capacity. The World Anti-Doping Agency has prohibited the use dustats in sports (WADA, list S2.1) [2]. WADA set the limit of detection of dustats in samples at 2 ng/mL [3]. The screening of dustats in formulations and biological fluids (urine, blood serum/plasma) is usually carried out by high performance liquid chromatography coupled with mass spectroscopy (HPLC–MS) [4]. Other dustat screening methods such as gas chromatography (GC), potentiometric titration, UV spectrophotometry, infra-red spectroscopy (IR), nuclear magnetic resonance spectroscopy (NMR), and activity and affinity-based fluorescence assays have been demonstrated in the literature [5, 6]. These methods are usually time-consuming, require complex sample preparation procedures, and expensive equipment. Additionally, the sample matrix can obscure the screening result, thus undermining the accuracy and sensitivity of the analytical method. Therefore, there is a need to develop cost-effective, rapid, sensitive, and selective methods and sensors for the screening of HIF inhibitors in clinical and doping control samples.

Surface-enhanced Raman spectroscopy (SERS) is a powerful analytical technique that significantly enhances the Raman scattering signals of molecules that are adsorbed on metallic nanostructures. SERS offers high sensitivity, label-free detection, and rapid analysis for a wide range of molecules in various applications. In SERS, the Raman signal is intensified due to the combination of two enhancement mechanisms: electromagnetic and chemical [7]. The electromagnetic mechanism involves the local enhancement of electromagnetic fields by metal nanostructures (e.g. gold, silver, copper), which amplifies the Raman scattering signals of nearby molecules. The chemical enhancement is due to the adsorption of the analyte molecule to the metal nanostructure surface and the redistribution of the molecular energy levels near the Fermi level of the metal. This process leads to the formation of a charge-transfer electronic state that, when excited by a matching energy from a laser excitation source, an intensified Raman signal is generated. Various types of SERS materials have been demonstrated in the literature including 3D metallic colloids, membrane substrate [8, 13], 2D substrates [9], and magnetic substrates [10, 11]. Due to the high enhancement in the SERS measurement, it can be used to quantify extremely low concentrations of biomolecules such as doping agents [12].

In this work, low-cost plasmonic nickel foam was synthesised as a highly sensitive sensor for SERS measurements. The nickel foam is a lightweight three-dimensional porous polystyrene framework that is coated with nickel film [13, 14]. Closely packed forests of cauliflower-shaped gold nanostructures were deposited onto the foam using a simple chemical deposition method, to create an abundance of hotspots for sensitive SERS measurements [15]. The synergistic charge transfer effects of the gold and nickel metals and the coupling between their surface plasmons contributed to the high enhancement in the SERS measurements by the synthesised SERS sensor [16, 17]. The new sensor was utilised for the screening of Molidustat and Vadadustat PHD2 inhibitors in aqueous solutions down to 0.01 µg/L, thus confirming the sensor exceptional sensitivity. The plasmonic nickel foam material was also used to synthesise a target-specific extractor chip for the extraction and purification of the target drugs from aqueous solutions and biological fluids. Prolyl hydroxylase domain-containing protein 2 (PHD2) recognition molecules were attached to the plasmonic nickel foam material using deep UV LED light as a green surface functionalisation process. The light reduces the disulphide bonds within the PHD2 molecule and generate active sulfhydryl groups (SH) that attach to the material surface via Au–S bonds. The new extractor chip was utilised to extract/purify Molidustat and Vadadustat from spiked saliva prior to SERS screening by the new sensor.

## Experimental

### Materials, reagents, and instrumentation

2-Quinolinethiol 97%, gold (III) chloride trihydrate (99.9%), sodium borohydride (98.0%), phosphate buffer saline (PBS) tablets, bovine serum albumin (BSA), and acetaminophen were purchased from Sigma-Aldrich (USA). Acetylsalicylic acid was provided by UNILAB (India) and loratadine was obtained from Sigma-Aldrich (USA). Dimethyl sulfoxide (DMSO) was purchased from Supelco (USA). Nickel foam was supplied by MTI Corporation (USA). Molidustat and Vadadustat were purchased from Millennium Science (Australia) and the recombinant PHD2 (EGLN1) protein was purchased from Active Motif (USA). Deionised (DI) water (18.2 MΩ cm at 25 °C) was utilised to prepare aqueous solutions.

Raman measurements were conducted using a handheld Raman spectrometer from Ocean Optics (USA). The spectral resolution of the spectrometer was 12 cm^−1^. A 785-nm NIR laser source was used for sample excitation (laser power = 5 mW at the sample). The measurements were carried out using the raster orbital scanning mode (ROS) to acquire an average Raman signal from the probed sample [18]. Raman spectra were acquired within the wavelength range of 500–1900 cm^−1^. Each sample measurement was repeated three times (*n* = 3) and each measurement was carried out using 10 accumulations (accumulation time = 100 ms, total acquisition time = 300 ms). The instrument software algorithm (OceanView Spectroscopy 1.5.07) was operated to automatically correct the background noise and fluorescence within the measurements.

### Fabrication and characterisation of SERS sensor

Rectangular pieces of nickel foam (dimension = 7 mm W × 12 mm L) were cut and placed into clean glass beakers that contain 30 mL of a 1:1:1 (v/v/v) deionised water:ethanol:acetone, respectively, and sonicated in an ultrasonic bath for 10 min to thoroughly clean the foam. The foam was then rinsed with DI water and dried by a stream of nitrogen gas.

To deposit the gold nanostructures onto the clean nickel foam, a chemical deposition method was used. The foam was placed into a glass vial that contained 3 mL of 4 mM HAuCl_4_ solution and sonicated for 10 s to remove the air bubbles that were trapped within the porous structure of the foam. The foam was kept in the solution for 30 min. To deposit gold nanostructures onto the nickel foam, 25-µL aliquots of 0.01% NaBH_4_ reducing agent were added dropwise every 2 min for 16 min. The process was repeated twice to increase the foam surface coverage with gold nanostructures. The foam was then removed from the HAuCl_4_ solution, washed with deionised water, dried under a stream of nitrogen gas, and stored for future use. The size, morphology, and coverage of the deposited Au nanostructures on the nickel foam were characterised by scanning electron microscope (SEM) using a Zeiss Sigma field emission scanning electron microscope (Germany). The elemental composition of the gold-coated nickel foam was confirmed using the energy dispersive X-ray spectroscopy (EDS) detector.

### Determination of the enhancement factor (EF), effect of gold nanostructure loading, and reproducibility of the SERS sensor

To determine the enhancement factor (EF) of the new SERS sensor, 2-quinolinethiol (2-QT) was used as a Raman probe. A stock solution of 2-QT (1 × 10^−2^ M) was prepared by placing 8.07 mg of the Raman probe into a 5-mL volumetric flask and adding 99% ethanol solvent to the mark. A total of 1 × 10^−7^ M solution of 2-QT was prepared by serial dilution using ethanol. Fifty microlitres of the 1 × 10^−2^ M 2-QT solution was placed on clean, bare nickel foam and left to dry for 10 min. The foam was then washed with DI water, dried under a constant stream of nitrogen, and screened using the handheld Raman spectrometer. Fifty microlitres of the 1 × 10^−7^ M 2-QT solution was placed on the SERS sensor and left to dry for 10 min. The SERS sensor was then washed with DI water, dried under a constant stream of nitrogen, and screened using the handheld Raman spectrometer. The EF was calculated using Eq. 1 below:

1 $$\text{EF}= \frac{ {I}_{\text{SERS}}}{{I}_{\text{Raman}}}x \frac{{C}_{\text{Raman}}}{{C}_{\text{SERS}}}$$

where *I*_SERS_ is the Raman signal intensity of 2-QT @ 1370 cm^−1^ on the SERS sensor. *I*_Raman_ is the Raman signal intensity of the 2-QT @ 1370 cm^−1^ on the bare nickel foam. *C*_Raman_ is the concentration of the 2-QT solution (1 × 10^−2^ M) that was loaded onto the bare nickel foam. *C*_SERS_ is the concentration of the 2-QT solution (1 × 10^−7^ M) loaded onto the SERS sensor.

To identify the effect of gold nanostructure loading on the Raman signal enhancement of the SERS sensor, three pieces of the nickel foam (of equal dimensions) were coated with single, double, and triple layers of gold nanostructures to produce three SERS sensors. The fabricated sensors were then loaded with 10 µL of 1 × 10^−7^ M 2-QT solution and allowed to stand for 10 min at room temperature. The sensors were then rinsed with DI water, dried under a gentle stream of nitrogen gas, and screened by the handheld Raman spectrometer. A bare nickel foam of similar dimensions was also loaded with 10 µL of 2-QT (1 × 10^–2^ M), allowed to stand for 10 min at room temperature. The foam was then rinsed with DI water, dried under a gentle stream of nitrogen gas, and screened by the handheld Raman spectrometer. The EF of three SERS sensors was calculated using Eq. 1.

A gold-nanostructured paper SERS substrate was fabricated using the chemical deposition method. Three millilitres of 4 mM HAuCl_4_ solution was placed onto filter paper and left to sit for 30 min; then, 25-µL aliquots of 0.01% NaBH_4_ reducing agent were added dropwise every 2 min for 16 min. The paper was washed with DI water and dried under nitrogen. The process was repeated twice. The fabricated paper SRS substrate was loaded with 10 µL of 2-QT (1 × 10^−7^ M), allowed to stand for 10 min at room temperature, rinsed with DI water, and dried under nitrogen gas. A bare paper substrate (not coated with gold nanostructures) was loaded with 10 µL of 2-QT (1 × 10^−2^ M), left to stand for 10 min, then rinsed, and dried. The paper substrates were screened by the handheld Raman spectrometer and the EF was calculated using the Raman signal intensity at 1372 cm^−1^ and Eq. (1).

To assess the reproducibility of the EF of the new SERS sensor, 50-µL aliquots of 1 × 10^−7^ M 2-QT were loaded onto 12 SERS sensors that were prepared by depositing two layers of gold nanostructures onto 12 pieces of nickel foam rectangular pieces of equal dimensions using the described chemical deposition method. The sensors were washed with DI water and dried under nitrogen gas. The prepared SERS sensors were then loaded with 10 µL of 1 × 10^−7^ M 2-QT solution, allowed to stand for 10 min at room temperature, rinsed with DI water, dried under a gentle stream of nitrogen gas, and screened by the handheld Raman spectrometer. To determine the stability and shelf life of the new SERS sensor, 10 µL of 1 × 10^−7^ M 2-QT was loaded onto the sensor and allowed to stand for 10 min. The substrate was then rinsed with deionised water, dried, and screened by the handheld Raman spectrometer after 1, 7, and 14 days.

### Preparation of stock and working solutions of Molidustat and Vadadustat

Stock solutions of Molidustat (5020 µg/L) and Vadadustat (1993.5 µg/L) were prepared by dissolving an appropriate weight of the drug in 1 mL of DMSO. One hundred microlitres of the drug stock solution was transferred into a clean vial and diluted to 1 mL using ethanol (99% v/v). The final concentration of the Molidustat and Vadadustat dilute solutions was 502 µg/L and 199.35 µg/L, respectively. Aliquots of the resulting drug solutions were further diluted with ethanol to obtain drug solutions in the concentration range of 100 to 0.05 µg/L.

### Determination of Molidustat and Vadadustat by the SERS sensor

The Raman spectra of the Molidustat and Vadadustat were acquired for the first time using the new SERS sensor. Twenty-five microlitres of the 100 µg/L drug solution was loaded onto the SERS sensor and left to stand for 15 min. The sensor was then dried under a stream of nitrogen gas and screened by the handheld Raman spectrometer (*n* = 3).

For the quantification of Molidustat and Vadadustat by SERS, 25-µL aliquots of 100 µg/L, 50 µg/L, 10 µg/L, 5 µg/L, 1 µg/L, 0.5 µg/L, 0.1 µg/L, 0.05 µg/L, and 0.01 µg/L drug solutions were loaded onto SERS sensors, left to stand for 15 min, and then screened by the handheld Raman spectrometer. The relationship between the Raman signal intensity at 676 cm^−1^ (for Molidustat) and 995 cm^−1^ (for Vadadustat) was plotted against the drug concentration to develop a calibration plot of each drug. The limit of detection (LOD) of each drug was calculated using the equation:

2 $$\text{LOD }= 3.3 \sigma / m$$

where *σ* is the standard deviation of the blank and *m* is the slope obtained of the calibration curve.

### HPLC–MS measurements

HPLC–MS was used to screen 0.01 µg/L and 100 µg/L standard solutions of Molidustat and Vadadustat. The measurements were carried out using a Dionex UltiMate 3000 UHPLC system. Ten microlitres of the drug standard solution was injected onto a C18 HPLC column (Phenomenex Luna, 5 µm, 100 Å, 250 × 2.0 mm). The column temperature was maintained at 40 °C. The mobile phase consisted of 5 mM aqueous ammonium acetate (solvent A) and acetonitrile (solvent B). Isocratic elution was carried out using 20% B for 0.6 min. This was followed by a linear gradient elution to 95% B over 7 min. The mobile phase composition was held at 95% B for 3 min before re-equilibration to 20% B. The flow rate of the mobile phase was kept at 0.4 mL/min. For the simultaneous monitoring of analytes, the eluate was passed through a UV diode array detector that was set at 214, 254, 280, and 360 nm wavelengths. The eluate was then passed into a Q Exactive Plus high-resolution Orbitrap mass spectrometer (Thermo Fisher Scientific, Bremen, Germany) that has a heated electrospray ionisation (HESI) source operating in the positive ion mode. A spray voltage of 3.0 kV was applied to the quartz silica capillary of the ion source to generate a fine spray of charged droplets. The temperature of the capillary was maintained at 320 °C. Nitrogen was used as both the sheath gas (flow rate = 30 arbitrary units) and auxiliary gas (flow rate = 10 arbitrary units). The radio frequency (RF) level of the S lens of the MS spectrometer was set to 60, and the auxiliary gas heater temperature was set at 150 °C. Spectra were acquired at a nominal mass resolution power of 70,000 that is measured at a mass-to-charge ratio (*m*/*z*) of 200.

### Fabrication of target-specific extractor chip for Molidustat and Vadadustat

A total of 0.023 mL of a 0.2 µg/L (4.3 µM) PHD2 stock solution was diluted with 10 mL of 1 × PBS (pH = 7.4) to a final concentration of 0.01 µM.

To fabricate a target-specific extractor chip for Molidustat and Vadadustat, nickel foam was coated with a double coat of gold nanostructures. The foam was then placed in a glass vial, 50 µL of the 0.01 µM PHD2 solution was added, and the vial was exposed to a 280-nm deep UV LED light beam for 30 min to activate the disulfide bonds within the PHD2 molecules and generate free sulfhydryl (SH) terminal groups that attach to the foam surface via Au–S bonds and form a mono layer of the recognition molecule [19]. The 280 nm deep UV LED beam was generated by an LED light source that is mounted on a metal-core printed circuit board (MCPCB-Mounted LED light model M280D4, Thorlabs, USA). The deep UV beam was kept at 75% of its full intensity, using the T-Cube LED Driver (LEDD1B, Thorlabs, USA) that is set at 0.35 A. The deep UV LED light source was connected to the LED driver using an LED connection cable (CAB-LEDD1, Thorlabs, USA). An image of the experimental setup of the light source is depicted in Fig. S1 in the supplementary document. The vial was moved away from the light source after 30 min and kept in the dark for 90 min. The foam was then removed from the glass vial, washed with 1 × PBS (pH = 7.4), and dried under a stream of nitrogen gas. To backfill any remaining unoccupied gold nanostructure surfaces on the foam, it was loaded with 50 µL of 0.1 µg/L bovine serum albumin (BSA), allowed to stand for 1 h, rinsed thoroughly with 1 × PBS (pH = 7.4), dried under nitrogen gas, and stored for future use.

### Selective binding of Molidustat and Vadadustat

Twenty-microlitre aliquots of Molidustat and Vadadustat standard solutions (drug concentration = 100 µg/L) were diluted with 1 × PBS solution (pH = 7.4) to a final volume of 100 µL. The final concentration of the drug (Molidustat or Vadadustat) in PBS was 20 µg/L. Two identical extractor chips were loaded with 50 µL of the Molidustat or Vadadustat solution (drug concentration = 20 µg/L) and allowed to stand for 15 min to bind the drug to the chip via the PHD2 recognition molecules [20]. The extractor chips were then rinsed with PBS (pH = 7.4) to remove any unbound molecules and dried under a gentle stream of nitrogen gas. The bound Molidustat and Vadadustat drug molecules were released and recovered by incubating the extractor chips in 50 µL of acidic PBS (1 × PBS pH = 2.4) for 15 min. Twenty-five-microlitre aliquots of the acidic PBS solution were then collected and loaded onto SERS sensors. The sensors were allowed to stand for 15 min, dried under a gentle stream of N2 gas, and screened using the handheld Raman device.

### Selectivity of the extractor chip

To confirm the selectivity of the extractor chip towards Molidustat and Vadadustat, an interference study was performed. Stock solutions of acetaminophen, acetylsalicylic acid, and loratadine (drug concentration = 1500 mg/L) were prepared by dissolving an appropriate amount of each drug into ethanol and diluting to 5 mL using 1 × PBS (pH 7.4). Serial dilutions were carried out using 1 × PBS (pH 7.4) to prepare 1500 µg/L aqueous standard solution of each drug. A negative control sample was prepared by adding 5-µL aliquots of the drug standard solution (concentration = 1500 µg/L) into a glass vial and the volume was completed to 1.5 mL using 1 × PBS solution (pH 7.4). The final concentration of each drug (acetaminophen, acetylsalicylic acid, and loratadine) was 5 µg/L. The concentration of Molidustat and Vadadustat drugs in the negative control sample was zero. A positive control sample that contained Molidustat was prepared by mixing 5-µL aliquots of acetaminophen, acetylsalicylic acid, and loratadine standard solution (concentration of each drug solution = 1500 µg/L) with 50 µL of Molidustat standard solution (concentration = 30 µg/L) into a glass vial and completing the volume to 1.5 mL. A positive control sample that contained Vadadustat was prepared by mixing 5-µL aliquots of acetaminophen, acetylsalicylic acid, and loratadine standard solution (concentration of each drug solution = 1500 µg/L) with 50 µL of Vadadustat standard solution (concentration = 30 µg/L) into a glass vial and completing the volume to 1.5 mL. The final concentration of acetaminophen, acetylsalicylic acid, and loratadine in the positive control samples was 5 µg/L and that of Molidustat and Vadadustat was 1 µg/L. Equal volumes of the negative and positive control samples were loaded onto independent extractor chips and allowed to stand for 15 min. The extractor chips were then rinsed with PBS (pH = 7.4) to remove any unbound drug and then dried under a stream of nitrogen gas. To recover the bound drugs from the extractor chips, each chip was incubated in a 50 µL aliquot of acidic PBS buffer solution (pH = 2.4) for 15 min. The extractor chip was then removed and the buffer solution loaded onto a SERS sensor, dried under a stream of nitrogen gas, and screened by the handheld Raman spectrometer.

### Determination of Molidustat and Vadadustat in a spiked saliva sample

Self-donated saliva was used in this experiment (saliva was donated by the author Rebecca Houston). The collection and use of the self-donated saliva was approved by the Queensland University of Technology ethics committee (approval number 8454).

The self-donated saliva was diluted one 100-fold using 1 × PBS (pH 7.4). Ten-microlitre aliquots of 50 µg/L Molidustat or Vadadustat standard solution were spiked in 240 µL of dilute saliva. The final concentration of the dustat drug in the spiked saliva was 20 µg/L. Extractor chips were incubated with 50-µL aliquots of the Molidustat-spiked saliva and Vadadustat-spiked saliva (concentration of the dustat drug = 20 µg/L) for 15 min to bind the drug to the chip via its PHD2 recognition layer. The extractor chips were then rinsed with 1 × PBS (pH 7.4) and dried under a gentle stream of nitrogen gas. To release and recover the bound drug, the extractor chips were incubated with 50-µL aliquots of acidic 1 × PBS buffer solution (pH = 2.4) for 15 min. The buffer solutions were then loaded onto SERS sensors and allowed to stand for 15 min. The sensors were then dried under a stream of nitrogen gas and screened using the handheld Raman spectrometer. The Raman signal intensities at 676 cm^−1^ (for Molidustat) and 995 cm^−1^ (for Vadadustat) were used to calculate the drug concentration in the spiked saliva sample.

## Results and discussion

### Synthesis and characterisation of SERS sensor

Gold nanostructures were deposited onto nickel foam by a chemical reduction method to develop a low-cost sensitive SERS sensor. Nickel was selected for the gold nanostructures deposition due to its magnetic property that can contribute to the enhancement effect within SERS measurements. The magnetic field of nickel increases the electromagnetic field around the deposited gold nanostructures, thus maximising the Raman signal amplification in SERS measurements [16, 21]. In addition, the synergistic charge transfer effects and plasmon coupling between the gold and nickel metals also contribute to a high Raman signal enhancement of analytes when they absorb and form a charge transfer complex on the bimetallic surface of the plasmonic nickel foam (the SERS sensor) [16, 17]. Further, the nickel foam provides a large surface area for the deposition of packed forests of gold nanostructures and an abundance of electromagnetic hotspots for high Raman signal enhancement [22].

The effect of the gold nanostructure loading on the EF in SERS measurements was studied by screening 2-QT on nickel foam substrates that were coated with single, double, and triple depositions of gold nanostructures. As shown by Fig. S2 (a), the bare and the gold-coated nickel foam showed no Raman vibration modes, thus indicating they are clean surfaces. Figure S2 (b) shows the increase in the 1370 cm^−1^ Raman signal intensity of 2-QT when the number of cycles for gold nanostructures deposition was increased from 1 to 3 cycles. The EF of the SERS sensor increased from 4.56 × 10^6^ to 1.09 × 10^7^ and 2.88 × 10^7^ when the nanostructures deposition was repeated once, twice, and three times, respectively. The increase in the EF value is attributed to the increased load of the gold nanostructures on the nickel surface. The high EF by the new SERS sensor was comparable to those manufactured by sophisticated lithography methods such as the gold-coated silicon nano pillar sensor [23].

To confirm the impact of the magnetic property of the nickel metal foam on the EF of the gold-nanostructured nickel foam material, gold nanostructures were deposited on a filter paper (non-magnetic surface), using the reported chemical deposition method, and 2-QT was used as a Raman probe to establish the EF of the paper SERS substrate (Fig. S3). The EF of the non-magnetic paper SERS substrate was found to be 1.60 × 10^6^ which is less than that of the gold-nanostructured nickel foam substrate (2.88 × 10^7^). This result confirms the contribution of the nickel magnetic property and the 3D porous structure to the high EF of the new substrate.

Although the triple deposition of gold nanostructures provides a high EF, double deposition is more cost-effective. In addition, the excessive gold deposition using three deposition cycles could lead to surface aggregation and reduced hotspot uniformity [24]. Therefore, the new SERS sensor was produced using two cycles of gold nanostructures on nickel foam.

**Fig. 1:**
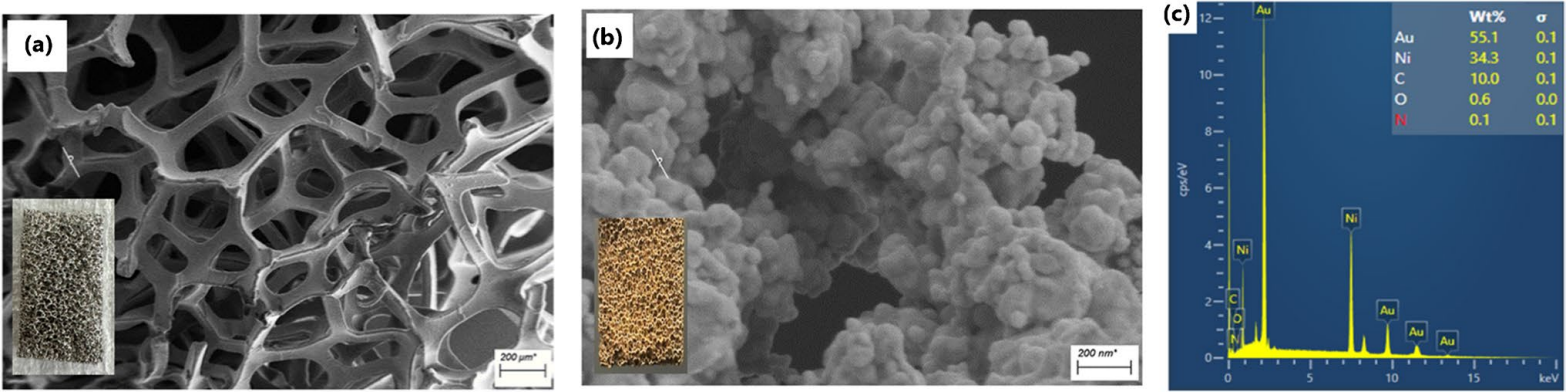
Surface analysis of the nickel foam before and after deposition of gold nanostructures. **a** SEM image of bare nickel foam at 200 µm magnification (the inset depicts a photograph of the uncoated foam). **b** SEM image of gold-coated nickel foam at 200 nm magnification (the inset depicts a photograph of the gold-coated foam). **c** EDS spectrum of the gold-coated nickel foam

The morphology of the gold nanostructures on the SERS sensor was investigated by scanning electron microscopy (SEM). Figure 1a and b depict the SEM image of the nickel foam before and after the deposition of the gold nanostructures. As shown by Fig. 1b and c, closely packed cauliflower-shaped gold nanostructures were deposited on the nickel foam with a weight percentage of 55.1%, thus creating an abundance of hot spots for sensitive SERS measurements.

**Fig. 2:**
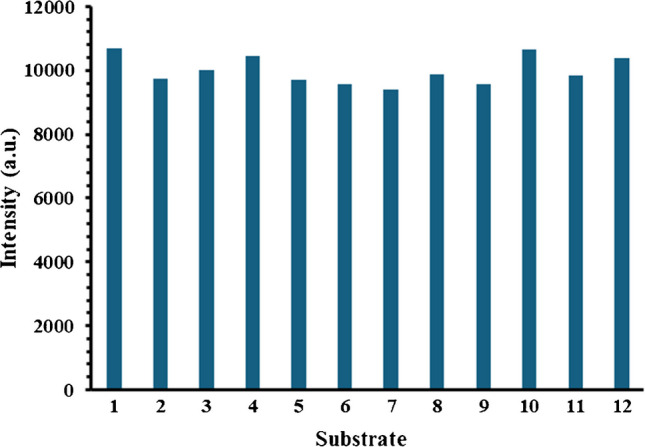
Repeatability assessment of 2-QT on different substrates

To confirm the reproducibility of the synthesised SERS substrate, 12 SERS sensors were loaded with equal volumes of 1 × 10^−7^ M 2-QT solution and screened by the handheld Raman spectrometer. As indicated by Fig. 2, the signal intensity of the 2-QT Raman band @ 1370 cm^−1^ changed slightly between the sensors, and the relative standard deviation (RSD) between the SERS measurements of 2-QT was 4.43%, thus indicating the high reproducibility of the sensor’s synthesis process.

The shelf life of the new gold-nanostructured nickel foam SERS sensor was investigated by loading 2-QT (1 × 10^−7^ M) and monitoring the change in its Raman signal intensity at 1372 cm^−1^ over 14 days (Fig. S4a-b). As shown by the figure, there was a slight reduction in the 1372 cm^−1^ signal intensity over that time which indicates the good stability of the new SERS sensor. This result can be attributed to the chemical stability of gold at normal storage conditions.

### Identification and quantification of Molidustat and Vadadustat by the SERS sensor

**Fig. 3:**
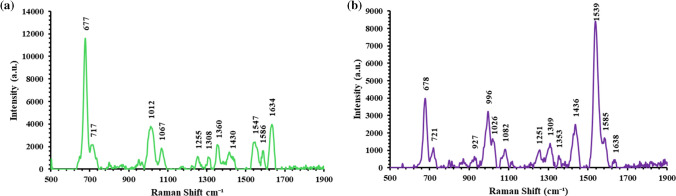
Raman spectra for **a** Molidustat and **b** Vadadustat by the new SERS sensor

The Raman spectra of Molidustat and Vadadustat were acquired for the first time by the new SERS sensor (Fig. 3a and b, Fig. S5). As shown by the Figure, the dustat drugs have a characteristic Raman band at 676 cm^−1^ that corresponds to Pyridine Ring breathing and triazole ring torsion (Table 1, Fig. S6). Vadadustat also shows a characteristic Raman band at 1537 cm^−1^ which can be attributed to C = C stretching in aromatic ring and C = N vibrations of the drug molecule (Fig. 3b). The Vadadustat Raman band at 997 cm^−1^ is attributed to N-Cα-C and C-N–N vibrations.

**Table 1:** Band assignments for the SERS peaks of Molidustat and Vadadustat

Raman shift (cm^−1^)	Molidustat	Vadadustat	Band assignment	Ref
1632	Y	Y	C = O stretching	[25, 26]
1591	Y	Y	Amide II, N–H bend, C-N stretch	[25]
1556	Y	N	C = C stretching in aromatic ring	[26]
1537	N	Y	C = C stretching in aromatic ring, C = N	[26]
1431	N	Y	C-H bending, C–C stretching	[27]
1417	Y	N	C–C stretching between carboxylic acid	[23]
1350	Y	Y	C–C stretching, ring deformation	[27]
1315	Y	Y	Amide III, C-N stretch, N–H bend	[25, 26]
1067	Y	N	C–C stretching	[27]
1025	N	Y	C–C-H bending, C–C-C bending	[26]
1013	Y	N	C–C-H bending	[26]
997	N	Y	N-Cα-C, C-N–N	[25, 26]
711	Y	Y	Ring breathing	[27]
676	Y	Y	Pyridine Ring breathing, Triazole ring torsion, COOH bend, NCCO bend, pyrrole ring deformation	[24, 28–30]

**Fig. 4:**
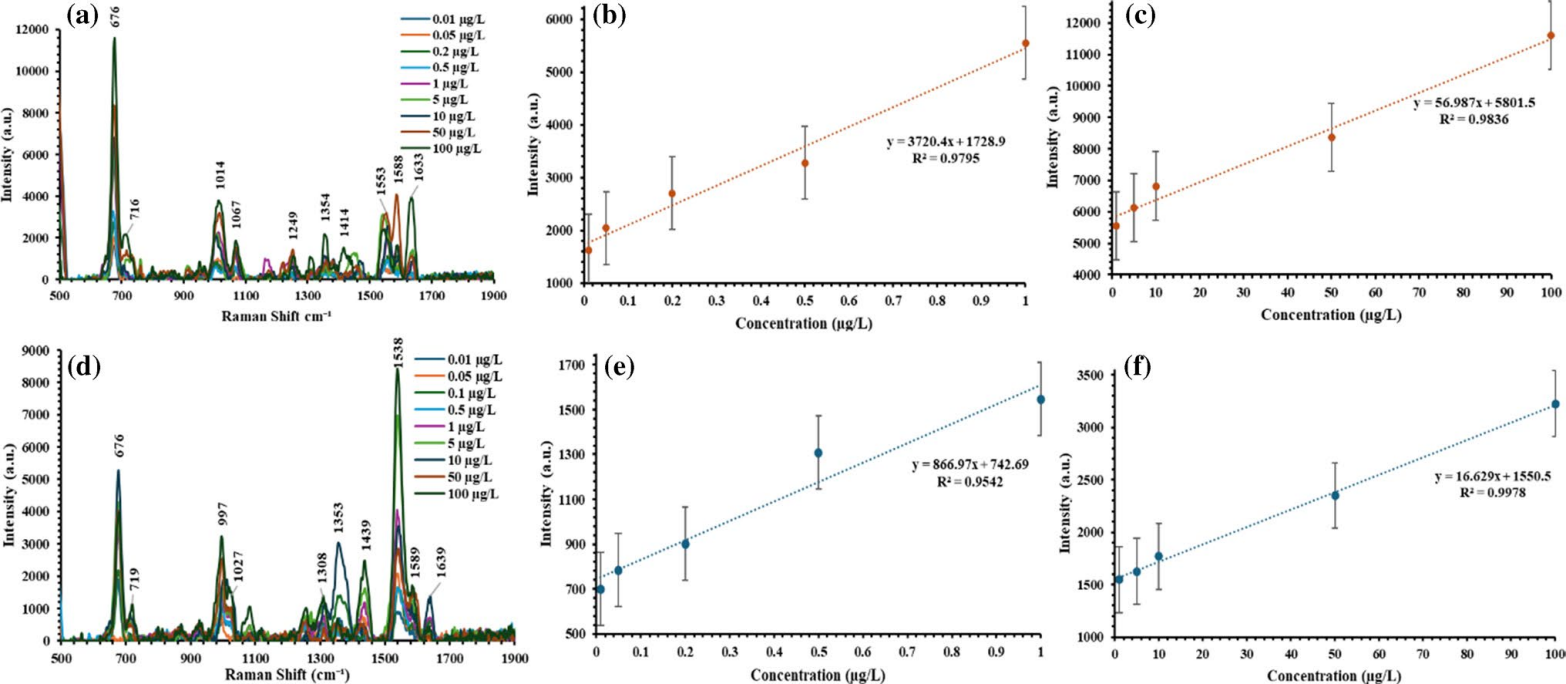
**a** SERS measurements of Molidustat at low and high concentrations (0.01–100 µg/L). **b** and **c** The calibration plots of Molidustat at 676 cm^−1^. **d** SERS measurement of Vadadustat at low and high concentrations (0.01–100 µg/L). **e** and **f** The calibration plots of Vadadustat at 997 cm^−^.^1^

The signal intensity of the Raman band at 676 cm^−1^ was found to change with the concentration of Molidustat (Fig. 4a). Therefore, it was utilised for the quantification of the drug within the concentration range of 0.01 to 100 µg/L. Calibration plots for the low (0.01–1 µg/L) and high (1–100 µg/L) concentration ranges of Molidustat are shown in Fig. 4b and c. The linear relationships between the Raman signal intensity @ 676 cm^−1^ and the drug concentration are represented by the regression equations: $y= 3720.4x +1728.9$ (*R*^2^ = 0.9795) and $y= 56.987x + 5901.5$ (*R*^2^ = 0.9836) for the low and high drug concentration ranges, respectively.

The Vadadustat Raman bands at 1537 cm^−1^ and 997 cm^−1^ were found to change with the concentration of the drug at the low and high concentration ranges (Fig. 4d). However, the change in the Raman signal intensity @ 1537 cm^−1^ did not follow a linear trend and could not be used for the drug quantification. To the contrary, the change in the Raman signal intensity @ 997 cm^−1^ followed linear trends that are represented by the regression equations $y=866.97x + 742.69$ (*R*^2^ = 0.9542) and $y= 16.629x +1550.5$ (*R*^2^ = 0.9978) for the low range and high concentration range of the drug, respectively (Fig. 4e and f). Using the calibration plots in Fig. 4b, c, e, and f and Eq. (2), the limits of detection (LOD) were found to be 0.004 µg/L for Molidustat and 0.01 µg/L for Vadadustat, respectively. The limit of quantification (LOQ) was determined experimentally by the calibration plot and found to be 0.01 µg/L for both drugs.

To qualitatively indicate the sensitivity of the SERS determination of the dustat drugs by the new sensor, the 0.01 µg/L and 100 µg/L standard solutions of Molidustat and Vadadustat were re-screened using HPLC–MS. As shown by Fig S7, the HPLC–MS method was able to detect the dustat drugs at high concentrations (100 µg/L). However, the HPLC–MS method could not detect low concentration (0.01 µg/L) of Molidustat. These results clearly indicate that the sensitivity of SERS sensor was similar or superior to that of HPLC–MS when applied for the screening of Molidustat and Vadadustat.

### Fabrication of target-specific extractor chip for the selective binding of Molidustat and Vadadustat

To develop a target-specific extractor chip that can selectively bind Molidustat and Vadadustat from complex matrices, a gold-nanostructured nickel foam substrate was functionalised with PHD2 as a target-specific recognition molecule for the dustat drugs. The surface functionalisation of the foam with PHD2 molecules was carried out using deep UV LED light @280 nm (Fig S1) in a green synthesis process that does not require the use of chemical reagents or reaction conditions that can compromise the bioactivity of the recognition molecule. The deep UV LED light reduces the disulfide bonds in the PHD2 molecule. These bonds are located between the cysteine residues 201 and 208 within the PHD2 molecule. The active site of PHD2 (the site that binds the dustat dugs) does not contain disulfide bonds. Therefore, the ability of the PHD2 to bind the dustat drugs is not affected by the light-induced reduction of its disulphide bonds [31, 32]. In this process, the deep UV LED light induces the electron transfer from excited tryptophan residues in the PHD2 molecule to its disulfide bond bridges. The gold nanostructures on the foam surface act as electron shuttles to facilitate the electron transfer, thus leading to the breakdown of the disulfide bonds and the generation of free sulfhydryl (SH) groups [33, 34]. The generated SH groups attach the PHD2 molecule to the gold nanostructures on the foam by forming stable Au–S bonds [35–37].

**Fig. 5:**
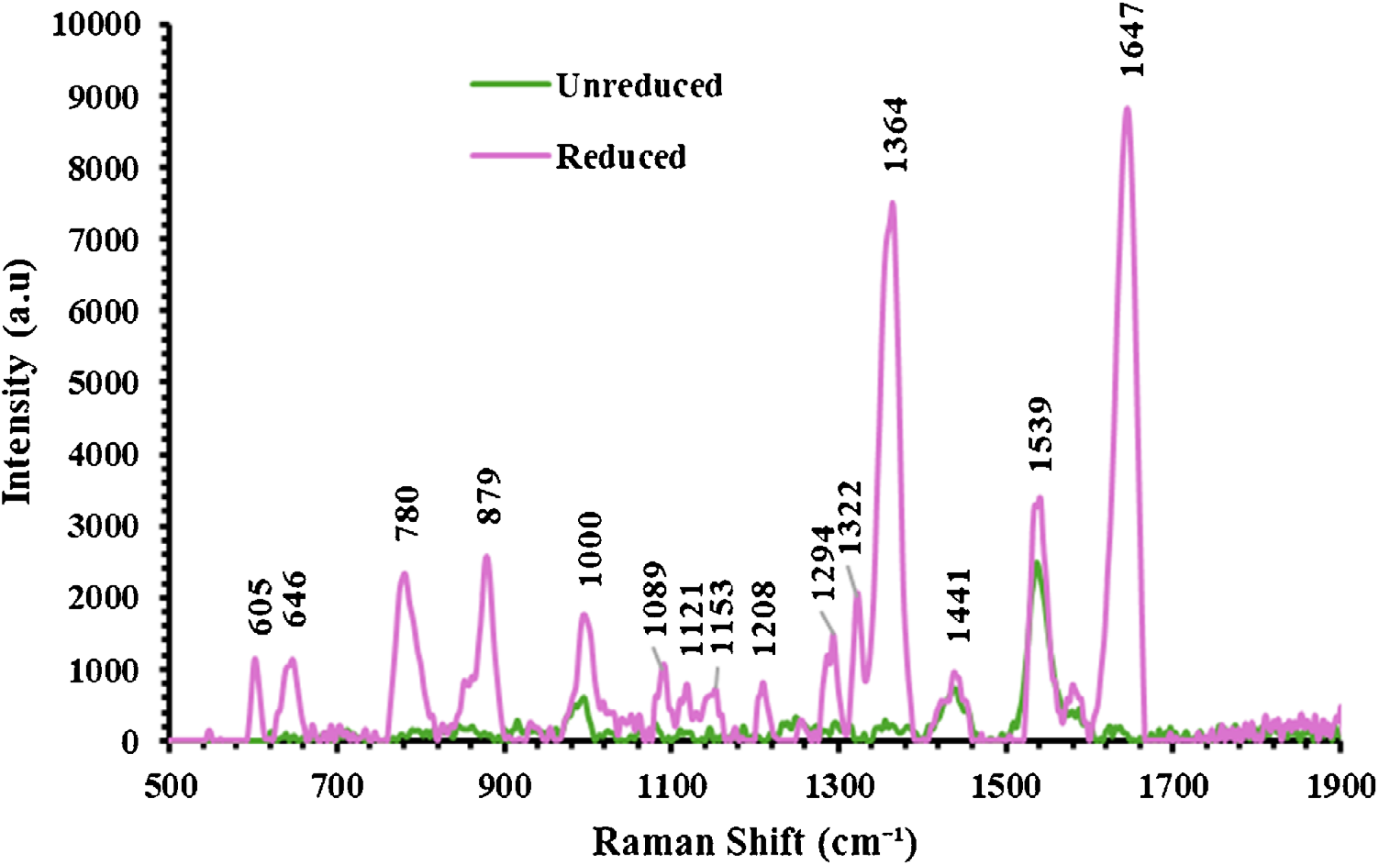
SERS measurements of un-reduced PHD2 molecules on the nickel foam and PHD2 molecules after light-induced reduction using deep UV LED light @ 280 nm and attachment to the plasmonic nickel foam

To confirm the light-induced attachment of the PHD2 to the surface of the gold-nanostructured nickel foam, the substrate was screened by SERS. As shown in Fig. 5, when PHD2 molecules were loaded onto the foam without exposure to the Deep UV light, they were poorly adsorbed and produced a very weak Raman spectrum in the SERS measurement. However, after the light-induced reduction and attachment to the gold nanostructures on the foam, the PHD2 exhibited noticeable Raman bands at 605 cm^−1^, 645 cm^−1^, 780 cm^−1^, and 879 cm^−1^ that are attributed to C–C-C-S stretching modes [38]. The band at 1364 cm^−1^ is assigned to the C-H ring bend vibration [38]. The Raman band at 1537 cm^−1^ can be attributed to the C-N and C–C stretching modes as well as in-plane vibrations of amino acid side chains within the molecule [39]. The Raman band at 1647 cm^−1^ is assigned to the amide I vibration mode [40, 41].

**Fig. 6:**
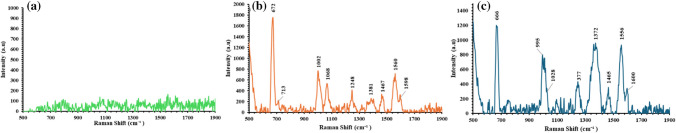
**a** SERS measurement of the extract of the negative control sample after extraction by the target-specific extractor chip. **b** and **c** The SERS measurements of Molidustat and Vadadustat after extraction from the positive control sample by the target-specific extractor chip

To evaluate the selectivity of the developed extractor chip, it was applied to bind/extract Molidustat and Vadadustat from negative and positive control samples. The bound drugs were released/recovered from the extractor chip using acidic PBS buffer (pH = 2.4). The buffer solution was then loaded onto the SERS sensor and screened by the handheld Raman spectrometer. Figure 6a shows that the characteristic Raman vibration modes of the Molidustat, Vadadustat, acetaminophen, acetylsalicylic acid, and loratadine drugs were absent in the Raman spectrum acquired from the negative control sample extract (Fig. S8 shows the reference Raman spectra of standard acetaminophen, acetylsalicylic acid, and loratadine on the gold nano-structured nickel foam substrate). This result clearly indicates that the extractor chip did not bind any of the other drugs that existed in the negative control sample. The result also indicates that the BSA backfilling agent successfully blocked the non-specific binding of the other drugs to the extractor chip. Figure 6b and c show that the Raman spectrum that was acquired from the extracts of the positive control samples contained characteristic Raman vibration modes of Molidustat and Vadadustat. These results confirm that the extractor chip was able to selectively bind and extract the target dustat drugs from their positive control samples.

### SERS determination of Molidustat and Vadadustat in aqueous solution and spiked saliva

**Fig. 7:**
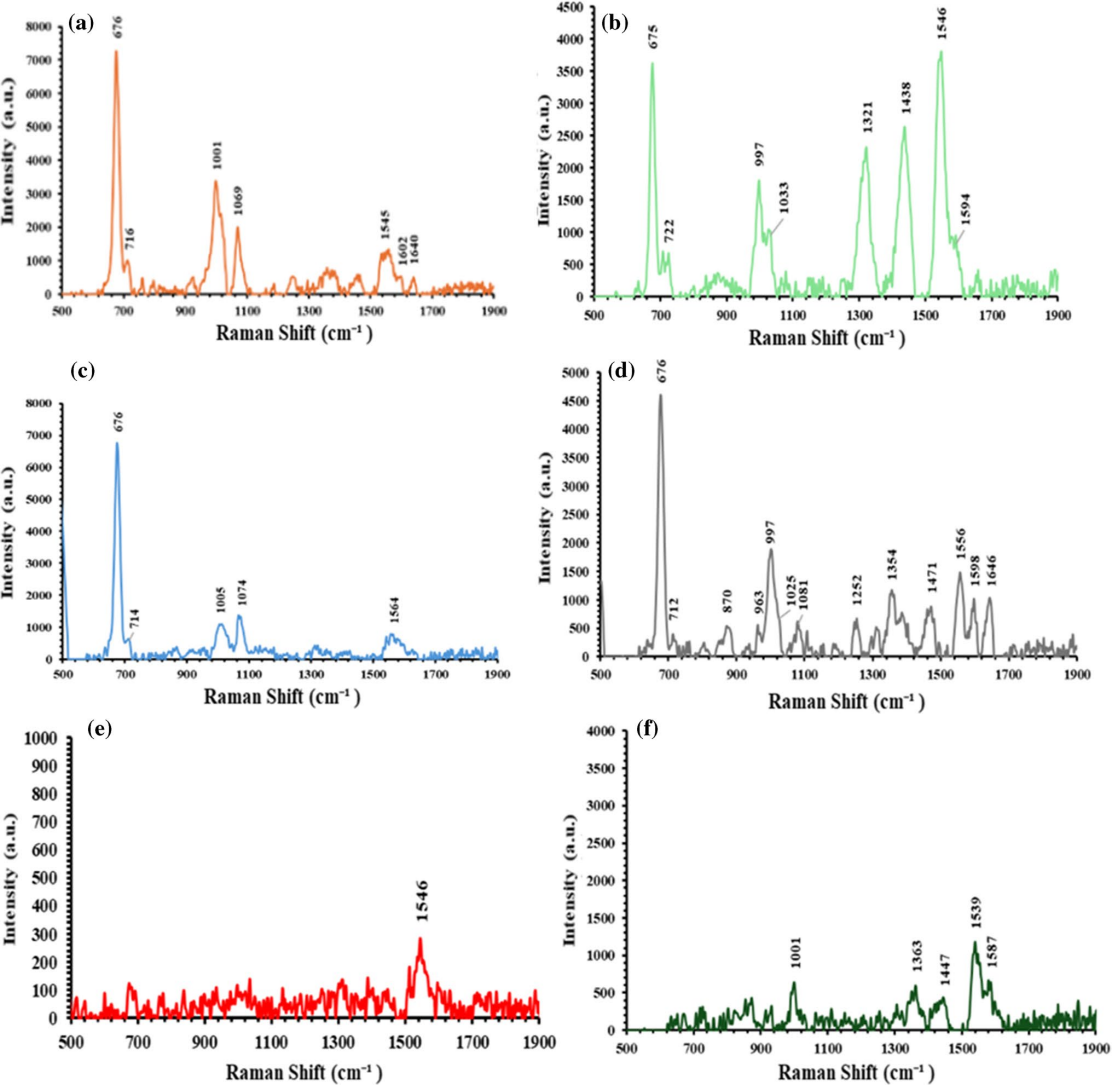
SERS measurements of Molidustat (**a**, **c**) after extraction/recovery from spiked aqueous samples and Vadadustat (**b**, **d**) after extraction/recovery from spiked human saliva samples. The dustat drugs were extracted by the target-specific extractor chip. The extracted drugs were released and recovered from the extractor chip using acidic PBS buffer solution (pH = 2.4). The extracts were loaded on clean SERS sensors and screened by the handheld Raman spectrometer. The SERS measurements of the acidic PBS buffer and blank saliva onto the SERS sensor are depicted in Fig. 7e and f, respectively

The extractor chip and SERS sensor were utilised for the quantification of the dustat drugs in aqueous solutions and biological fluids by SERS. Molidustat and Vadadustat were extracted from spiked aqueous and saliva samples using the extractor chip. The extracts were screened by SERS using the new sensor. Figure 7a–d depict the characteristic Raman vibration modes of Molidustat (@ 676, 711, 1005 cm^−1^) and Vadadustat (@ 676, 997, 1536 cm^−1^) that were observed in the Raman spectra of the extracts from the spiked aqueous and saliva samples. The slight variations in the 1354 cm^−1^ and 1471 cm^−1^ Raman band positions (Fig. 7b and d) can be attributed to the interaction between Vadadustat and the saliva matrix at the physiological pH environment. However, the Vadadustat characteristic bands at 676 cm^−1^ and 997 cm^−1^ were not affected and their positions were unchanged [42, 43]. The calibration plots in Fig. 4 were utilised to calculate the concentration of recovered Molidustat and Vadadustat in the extracts from the spiked aqueous solution and were found to be 18.7 µg/L and 19 µg/L for Molidustat and Vadadustat, respectively. The percentage of agreement between the actual concentration of the drug in the spiked sample and that screened by SERS was 93.5% and 95% for Molidustat and Vadadustat, respectively. Similarly, the concentration of the Molidustat and Vadadustat in the extract from the spiked saliva samples was found to be 18.3 µg/L and 21 µg/L, respectively. The percentage of agreement between the actual concentration of the Molidustat and Vadadustat in the spiked sample and that screened by SERS was 91.5% and 105%, respectively. The high percent agreement between the spiked and measured concentrations of the dustat drugs indicates the potential of the new SERS sensor and extractor chip materials for the rapid determination of drugs and doping agents for therapeutic drug monitoring and doping control applications.

This work reports the first SERS sensor for Molidustat and Vadadustat. The two-component design (extractor chip and SERS sensor) was chosen to ensure the high selectivity and sensitivity of the sensor (especially when used to screen complex biological matrices such as saliva). Our sensor directly detects the Raman spectrum of the target drug, thus maximising the integrity of the test and eliminating false positive results. SERS sensors that integrate sampling and detection have been demonstrated for biomolecule detection. These sensors are usually a complex screening mechanism and use a Raman reporter that indicates the presence of the target analyte [44]. Therefore, the integrated SERS sensing methods are usually indirect methods that report the Raman spectrum of the Raman reporter rather than that of the analyte itself. To the contrary, our SERS sensing method uses a highly selective recognition molecule (PHD2) to bind the target drug and a sensitive SERS sensor to directly detect the Raman spectrum of the drug itself.

Checkouri et al. recently demonstrated an LC–MS/MS method for the determination of Molidustat and Vadadustat in keratinised matrices [45]. The LC–MS/MS method requires lengthy sample preparation/analysis time and expensive equipment when compared to the new SERS method (Table S1). The LC–MS/MS method also uses toxic solvents for the LC mobile phase. Therefore, the new SERS sensor and method offer a cost-effective and sensitive approach for the rapid screening of dustats.

## Conclusion

We report new nanostructured materials that enables the selective binding and rapid SERS determination of Molidustat and Vadadustat drugs in spiked aqueous solutions and human saliva. The materials were developed using simple and green synthesis processes that are inexpensive, reproducible, and environmentally responsible. Closely packed gold nanostructures were deposited onto nickel foam to produce a new SERS sensor that provides a high enhancement factor of 1.09 × 10^7^ in SERS measurements. The new sensor was used to detect the Raman spectra of Molidustat and Vadadustat drugs for the first time and was utilised for their quantification at ultra-low concentration of 0.01 µg/L which is significantly lower than the 2 ng/mL WADA-recommended detection limit for these drugs in doping control practices. A novel light-driven surface functionalisation method was used to functionalise the surface of the gold-nanostructured material with PHD2 recognition molecules and synthesise a target-specific extractor chip for the selective binding of Molidustat and Vadadustat from aqueous solutions and human saliva. The new extractor chip and the new SERS sensor were utilised for the rapid determination of Molidustat in spiked aqueous and human saliva samples without the need for complex sample extraction procedure and sophisticated instrumentation. The new SERS sensor and extractor chip materials have strong potential for the rapid determination of drugs and doping agents in therapeutic drug monitoring and doping control applications.

## Supplementary Information

Below is the link to the electronic supplementary material.

ESM 1 (DOCX 1.12 MB)

## Data Availability

The authors declare that the data supporting the findings of this study are available within the paper and its Supplementary Information files. Should any raw data files be needed in another format they are available from the corresponding author upon reasonable request. Source data are provided with this paper.
