# Supplementary material for: Light-driven biosensor for the rapid and selective detection of hypoxia-inducible factor-prolyl hydroxylase domain inhibitors in aqueous media and saliva
Source: Mikrochim Acta. 2025 Oct 2;192(11):709. doi: 10.1007/s00604-025-07579-y (PMC12488750; doi:10.1007/s00604-025-07579-y)
Supplement: Supplementary file 1 — (DOCX 1.12 MB) [file 604_2025_7579_MOESM1_ESM.docx]

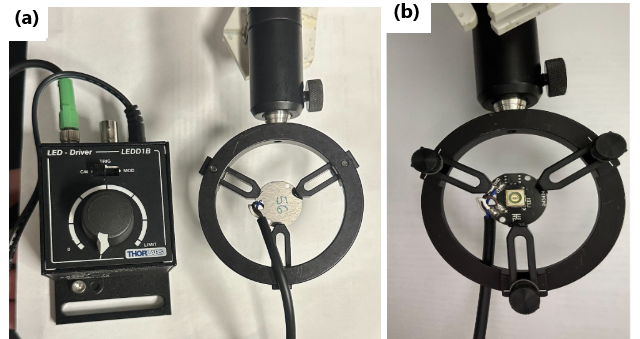


**Fig. S1** Images of the deep UV LED light source assembly from (a) back and (b) front view.


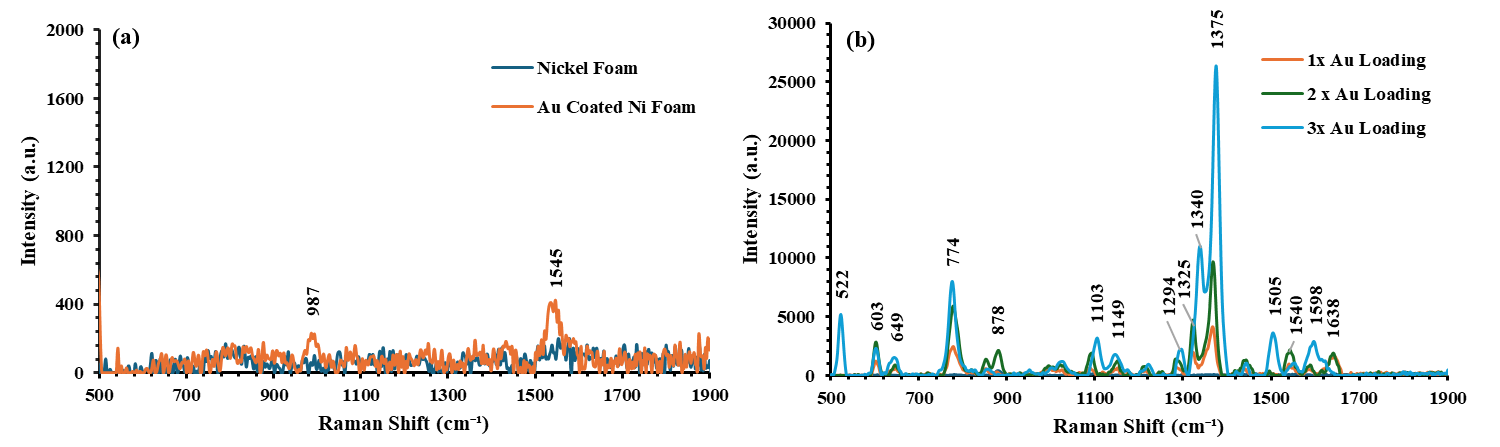


**Fig. S2** (a) SERS measurements of bare nickel foam (blue spectrum) and clean SERS sensor (orange spectrum), (b) SERS measurement of 2-QT (1x10^-7^ M) on SERS sensors. The orange green and light blue lines depict the spectrum of 2-QT on a signal, double and triple coat SERS sensors.


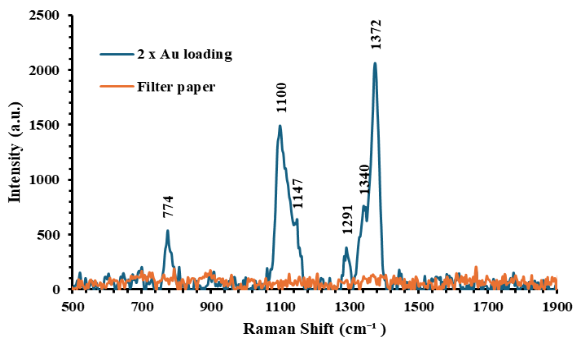


**Fig. S3** (a) SERS measurements of bare filter paper with 2-QT (1x10^-2^ M) (orange spectrum) and double gold coated filter paper sensor with 2-QT (1x10^-7^ M) (blue spectrum).


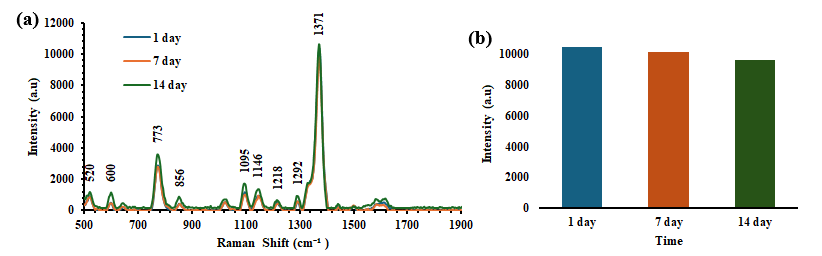


**Fig. S4** (a) SERS measurements of 2-QT (1x10^-7^ M) on SERS sensor after shelf storage of 1-, 7- and 14-days and (b) change in Raman signal at 1371 cm^-1^ after loading and shelf storage for 1-, 7- and 14-days.


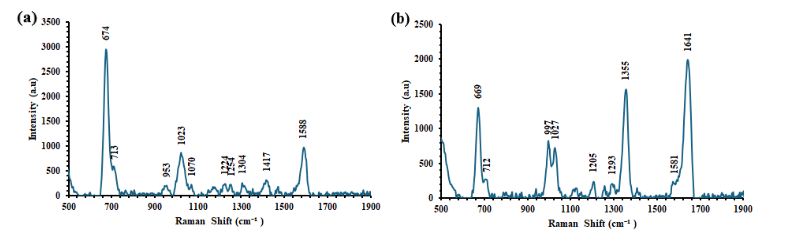


**Fig. S5** SERS measurements of (a) Molidustat (concentration =20 μg/L) and (b) Vadadustat (concentration =20 μg/L) on SERS sensor after 4 hours.


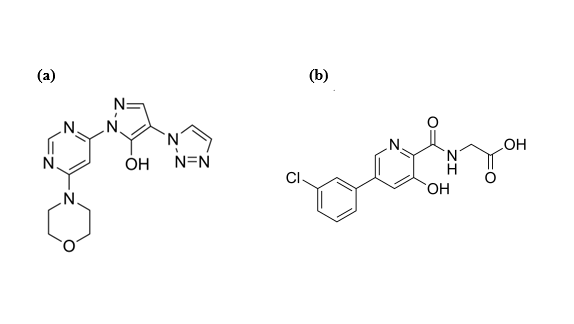


**Fig. S6** Molecular structure of (a) Molidustat and (b) Vadadustat


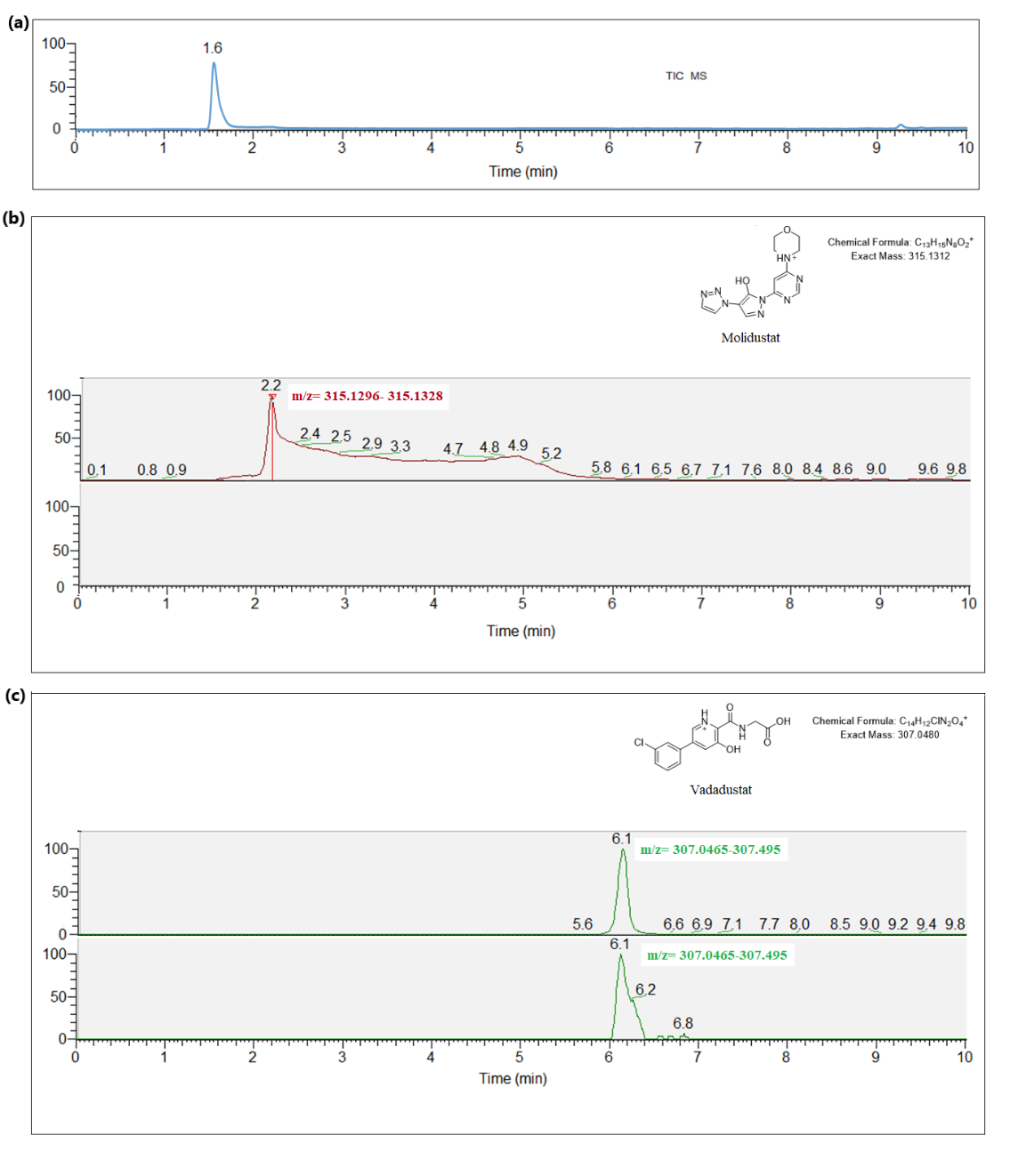


**Fig. S7** HPLC-MS measurements of (a) DMSO solvent, (b) 100 µg/L & 0.01 µg/L Molidustat standard solutions, (c) 100 µg/L & 0.01 µg/L Vadadustat standard solutions.


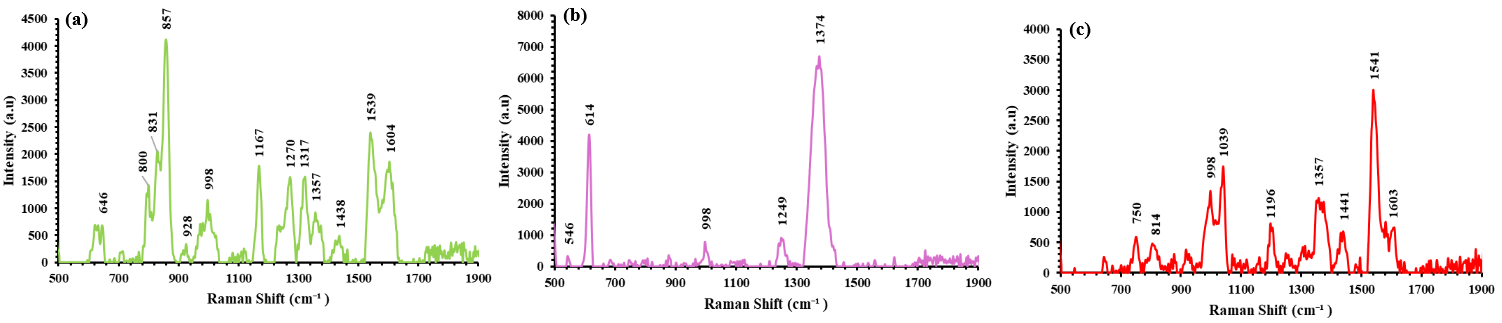


**Fig. S8** SERS measurements of (a) acetaminophen (b) acetylsalicylic acid (c) loratadine on Au-Ni foam.

**Table S1**: Comparison between SERS and HPLC-MS/MS methods for dustat detection.

| **Item** | **SERS Sensor (this work)** | **LC-MS/MS [1]** |
| --- | --- | --- |
| **Sample preparation** | 35 minutes (15 minutes binding, 5 minutes for releasing the bound drug, 15 minutes for loading onto the SERS sensor) | 16 hours |
| **Sensor Fabrication** | 45 minutes | Unknown |
| **Detection time** | 30 seconds | 3-6 minutes |
| **Equipment cost** | Handheld Raman spectrometer 32,000$ [2]  Nickel Foam (200 mm length x 300mm width x 1.6mm thickness), (56$ or 5.6$ for 7 mm W x 12 mm L SERS sensor)  [[3]](https://mtixtl.com/en-euea/products/bcnf-16m2) | LC-MS/MS (95,000$) [4]  HPLC C18 column (1340$) [5] |
| **Reagent cost** | Gold chloride,99% purity 1g = 277$ (only 3 mL of 4 mM gold chloride solution is required per SERS sensor)  Sodium Borohydride, 5g = 69$ (only 25 microlitre aliquots of 0.01% NaBH_4_ is required per SERS sensor) | Acetonitrile, HPLC-MS grade, 491$  Methanol, HPLC-MS grade, 120$ |
| **LOQ** | Molidustat: 0.01µg/L  Vadadustat: 0.01µg/L | Molidustat: 2pg/mg (2µg/L)  Vadadustat: 5pg/mg (5µg/L) |

References

1. Checkouri, A., Gheddar, L., Arbouche, N., Raul, J. S., & Kintz, P. (2024). Simultaneous detection of three hypoxia‐inducible factor stabilizers—molidustat, roxadustat, and vadadustat—in multiple keratinized matrices and its application in a doping context. Drug Testing and Analysis.

2. KWIPPED, I. (2016). IDRaman 785nm Raman spectrometer. KWIPPED.com. Retrieved 14/09/2025 from <https://www.kwipped.com/rentals/product/idraman-785nm-raman-spectrometer/19017>

3. Nickel Foam (200mm length x 300mm width x 1.6mm thickness) - bcnf-16m2. (2025). Retrieved 14/09/2025 from Mtixtl.com. https://mtixtl.com/en-euea/products/bcnf-16m2

4. Waters Xevo TQ-S micro For Sale. (2025). Labx.com. Retrieved 14/09/2025 from <https://www.labx.com/product-a/waters-xevo-tq-s-micro>

5. Acquity UPLC BEH C18 Column, 130Å, 1.7 μm, 2.1 mm X 100 mm. (2023). Waters.com. Retrieved 14/09/2025 from https://www.waters.com/nextgen/us/en/shop/columns/186002352-acquity-uplc-beh-c18-column-130a-17--m-21-mm-x-100-mm-1-pk.html

‌
